# Effects of optimal timed automatic awakening from a short daytime nap on cognitive performance, alertness, and fatigue

**DOI:** 10.1038/s41598-025-21008-3

**Published:** 2025-10-24

**Authors:** Yoko Suzuki, Chihiro Suzuki, Yurina Suzuki, Fusae Kawana, Tomohiro Ohigashi, Kazushi Maruo, Takahiro Watanabe, Takashi Abe

**Affiliations:** 1 Tsukuba Institute for Advanced Research (TIAR), International Institute for Integrative Sleep Medicine (WPI-IIIS), University of Tsukuba, 1-2 Kasuga, Tsukuba, 305- 0821 Ibaraki Japan; 2 Tsukuba Clinical Research & Development Organization, University of Tsukuba, Tsukuba, 305-8575 Japan; 3 Institute of Medicine, University of Tsukuba, Tsukuba, 305-8575 Japan; 4 KYOCERA Corporation, Kyoto, 612-8501 Japan

**Keywords:** Short nap, Sleep inertia, Fatigue, Blood flow meter, Cognitive performance, Wearable device, Psychology, Health care

## Abstract

Daytime napping improves performance, which is maximized with a post-N2 9-min nap. We evaluated whether a system that enables optimal-time automatic awakening using blood flow parameters could improve performance, sleepiness, and fatigue compared to no-nap. Additionally, we investigated whether its performance was comparable to manual awakening based on polysomnography. Eighty-one healthy adults (33.6 ± 12.8 years) were randomly assigned to automatic- or manual-awakening or rest groups. A task bout comprising a digit-symbol substitution test (DSST), visual detection test, and sleepiness and fatigue questionnaires was performed three times per session before napping and for six sessions after napping. In all post-nap sessions, sleepiness and fatigue in the automatic awakening group decreased, compared to the rest group, and were comparable to those in the manual awakening group. The DSST improved in the sixth post-nap session for the manual awakening group compared to the rest group; no improvement was observed in the automatic awakening group. The system model was refined by adding training data and tested on 50 healthy adults (40.6 ± 13.1 years). The test results revealed that the N2 detection accuracy of the system improved. The optimal automatic awakening system improves subjective sleepiness and fatigue, and further improvements in its accuracy may enhance post-nap performance.

**Supplementary Information:**

The online version contains supplementary material available at 10.1038/s41598-025-21008-3.

## Introduction

Epidemiological research indicates that daytime sleepiness affects 20–33% of the global population^1–3^ and adversely affects health, wellbeing, safety, cognitive function, and productivity^1,2,4,5^. Countermeasures against daytime sleepiness include bright light, blue light, caffeine intake, sleep extension, and daytime napping^6–10^. Among these, daytime napping has the following benefits. First, if administrative barriers or cultural resistance are overcome, it can be quickly introduced in the workplace or school^11–13^. Second, the positive effects of napping on health have been reported^14–17^, and the short-term effects include alleviation of neuroendocrine stress, improved immune function, improved cognitive performance, enhanced productivity, reduced sensitivity to pain, reduced nighttime sleepiness in night-shift workers, and a reduction in accidents owing to decreased sleepiness^14–17^. In addition, daytime napping can be safely used by expectant mothers and children^11,16–18^. Therefore, daytime napping may, immediately and safely, alleviate fatigue and improve alertness and cognitive performance.

However, long naps can induce several adverse effects. First, post-nap performance improvement may be inhibited because of sleep inertia, which is possibly caused by waking from slow-wave sleep (SWS)^15,19,20^. Sleep inertia was more likely to occur in naps with a duration ≥ 30 min because of the higher probability of reaching SWS^12,14,20,21^. Second, napping for too long may have negative longitudinal health consequences; a > 30-min nap duration is associated with increased risks of cardiovascular disease, diabetes, and metabolic syndrome^22–24^. Excessively long naps are potentially associated with poor performance and an increased risk of adverse health. Therefore, a balanced nap duration that maximizes task performance and minimizes sleep inertia needs to be identified.

Previous studies have examined the association of optimal nap duration with improved cognitive performance^15,21,25–27^. Comparisons of nap durations from 30 s to 30 min, evaluated against the no-nap condition, revealed that an at least 10-min nap improved alertness and cognitive performance^21,25,26^. Hayashi et al. (2005) showed that, compared to no naps, awakening 3 min after N2 improved cognitive performance^27^. Subsequently, Hayashi et al. (2014) compared task performance on awakening after 3, 6, 9, 12, and 15 min following N2 onset and found that performance was maximized under the 9-, 12-, and 15-min conditions^28^. Thus, it was posited that waking 9 min after N2 onset constitutes the optimum nap time to maximize performance and minimize sleep time^28^.

Short naps have been introduced in the workplace^12,13^; however, there are significant problems with optimal napping when individuals are awakened 9 min after N2 onset. First, it is difficult to predict the N2-onset timing and manually set the alarm in advance because of interindividual and inter-sleep variations in N2 timing. Second, polysomnography (PSG) is required to assess sleep parameters, and waking 9 min after N2 onset requires a technologist to evaluate the PSG and manually awaken the participant. Third, though photoplethysmography (PPG)-equipped wearable devices can ensure an optimal napping time and are easy to use for sleep measurement, these devices have several limitations: (a) the accuracy of commercial devices for sleep-stage determination must be improved^29^; (b) N2 detection is difficult because many devices determine N1 and N2 together as ‘light sleep’^29^; (c) few devices determine sleep stages in real time. These difficulties indicate that optimal napping is impossible in offices and other workplaces, and this highlights the need for new approaches.

**Fig. 1:**
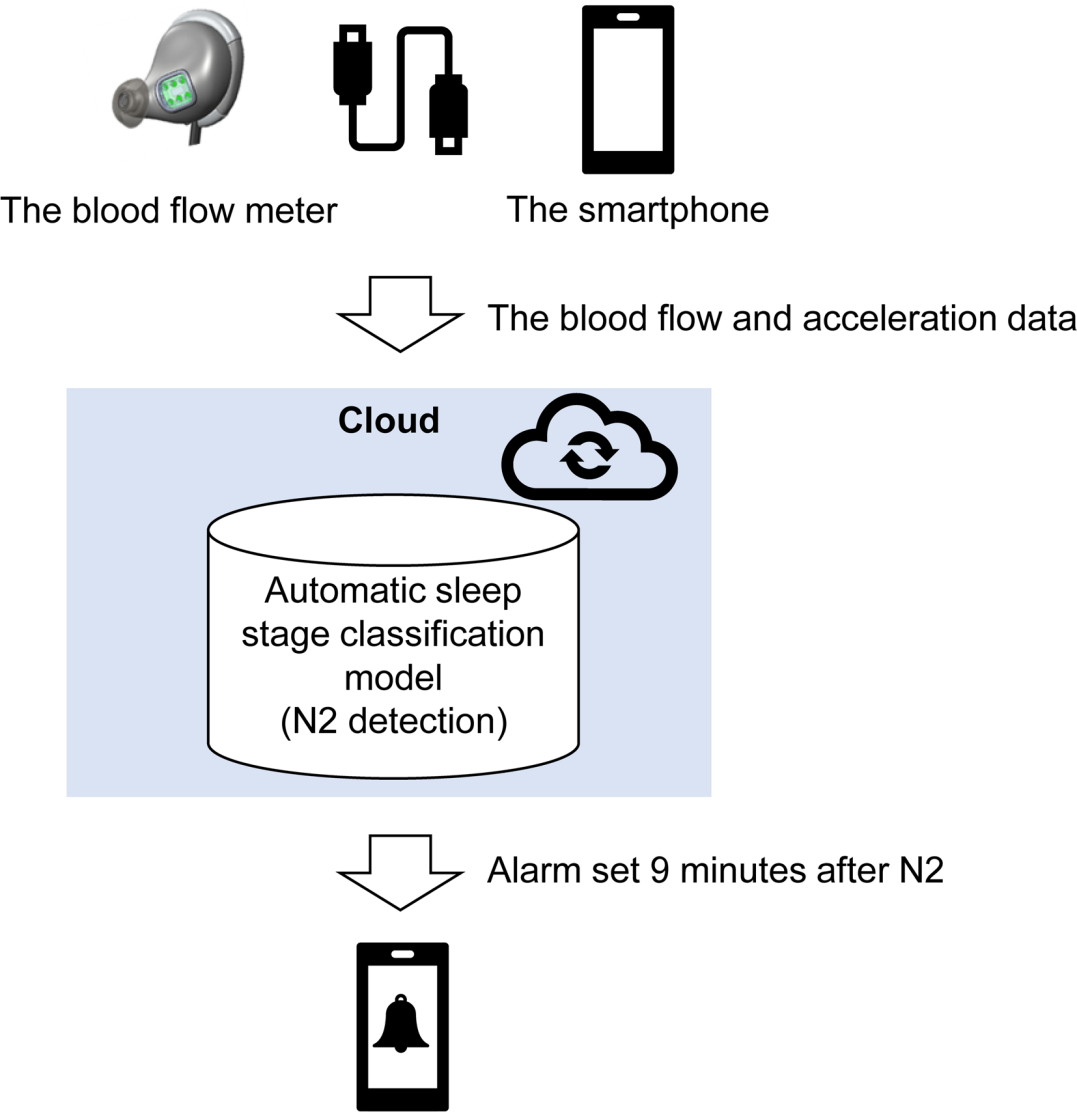
The autonomic awakening system. The automatic awakening system consisted of an application on a smartphone and an earphone-shaped blood flow (BF) meter. A Universal Serial Bus cable connects the BF meter to the smartphone. The BF and acceleration information from the meter were transmitted to the cloud via the application, and the sleep-stage scoring N2 model in the cloud was used to analyze the sleep stage. The smartphone alarm was set to go off 9 min after the N2 onset.

Therefore, we developed a system to automatically determine the sleep stage using an earphone-shaped blood flow (BF) meter and wake the participant 9 min after the detection of the first N2 (first-generation, Fig. 1). BF parameters measured using a laser Doppler method contain rich information^30^ and seem to be applicable for detecting N2. Based on this, we developed a method to automatically estimate N2 using BF data and to trigger an alarm 9 min after the first N2 (Fig. 1).

This study aimed to verify whether post-nap effects in the automatic awakening group were superior to those in the rest group and comparable to manual awakening based on real time PSG observation. Experiment 1 aimed to determine whether napping with an automatic awakening system improves performance and alleviates fatigue or sleepiness compared to no nap. In addition, we examined whether the automatic awakening system was comparable to manual awakening. We evaluated the accuracy of automatic N2 detection for the first-generation. The accuracy was calculated as follows: number of correct N2 detections/total number × 100. We assumed the correct N2 detection if the system detected N2 ± 6 min (target duration of 3–15 min after N2 to improve task performance^27,28^ or if no N2 was shown in the system and PSG, showing that the system had an accuracy of 25.9%. Considering the low accuracy, after the experiment, we added training data by performing simultaneous PSG and BF meter measurements during a nap (training data: *n* = 81; *n* = 120 each for the first- and second-generation prototype). In Experiment 2, the newly trained models and refined equipment were tested on 50 healthy adults. We validated the second-generation prototype system with a target accuracy of 80.0%.

## Results

### Experiment 1: Pre-experiment sleep–wake control

Data from 81 participants are summarized in Table 1, which shows a significant main effect of body mass index (BMI) on the group (Table 1). Post-hoc analysis revealed a significant difference in BMI between the rest and manual awakening groups (*p* = 0.011). There was no other significant difference in BMI (automatic vs. manual, *p* = 0.304; rest vs. automatic, *p* = 0.122). Other biological indicators and questionnaires showed no significant intergroup differences (Table 1).

**Table 1:** Characteristics of the participants in Experiment 1.

	Total (*N* = 81)	Automatic (*N* = 27)	Manual (*N* = 27)	Rest (*N* = 27)	*P*-value
Sex, n, male/female	34/47	11/16	9/18	14/13	0.423
Age, years	33.6 ± 12.8	33.1 ± 13.1	33.9 ± 12.4	34.0 ± 13.3	0.832
BMI, kg/m^2^	20.7 ± 1.7	20.6 ± 1.5	20.1 ± 1.6	21.3 ± 1.9	0.038
MEQ-score	53.5 ± 7.0	52.7 ± 7.3	53.0 ± 8.3	54.7 ± 5.3	0.470
MEQ-type, n, Morning/Intermediate/Night	23/57/1	6/20/1	10/17/0	7/20/0	0.552
PSQI-score	3.3 ± 1.3	3.5 ± 1.0	3.1 ± 1.3	3.3 ± 1.5	0.701

BMI, body mass index; MEQ, Morningness–Eveningness Questionnaire; PSQI, Pittsburgh Sleep Quality Index.

No significant intergroup difference was observed in the pre-experimental sleep time recorded using Fitbit (*p =* 0.670). The sleep time in the rest, manual awakening, and automatic awakening groups was 390.6 ± 30.9, 399.9 ± 46.1, and 392.5 ± 42.7 min, respectively.

### Experiment 1: naptime sleep indices and subjective sleep

The napping group reported no adverse events while using the BF meter. The accuracy of the automatic N2 detection system was calculated. We defined the first N2 as that reached N3 without awakening or that followed ≥ 9 min of consecutive sleep, showing that the automatic awakening system had an accuracy of 25.9%. Next, the accuracy of the first N2 was evaluated according to the American Academy of Sleep Medicine (AASM) criteria^31^, and remained unchanged at 25.9%. Contrary to our expectations, the automatic awakening group had significantly longer sleep variables than the manual awakening group for total recording time, total sleep time (TST), and N1, N2, and N3 (Table 2). The proportions of participants who reached N3 were 63.0% in the automatic awakening group and 7.4% in the manual awakening group. Sleep efficiency (SE) was significantly higher in the automatic awakening group than in the manual awakening group (Table 2). TST was 11.8 ± 3.9 min in the manual awakening group and 22.6 ± 6.5 min in the automatic awakening group (Table 2), and its standard deviation (SD) increased in the automatic awakening group. Levene’s test revealed that the variance was significantly greater in the automatic awakening group (*p* < 0.001, Supplementary Table S1). The time of N2 detection in a spindle or K-complex appearance was examined using offline PSG analysis, and the time until the alarm went off was calculated. The time from N2 to the alarm was 19.6 ± 6.0 min in the automatic awakening group, which was longer than the 9.6 ± 1.5 min in the manual awakening group. Table 2 summarizes the results of subjective sleep evaluations. There was no significant intergroup difference on subjective sleep evaluation (Table 2).

**Table 2:** Sleep-stage scoring and subjective sleep evaluation in Experiment 1.

	Manual	Automatic	*P*-value
TRT, min	16.7 ± 5.4	26.2 ± 5.9	< 0.001
TST, min	11.8 ± 3.9	22.6 ± 6.5	< 0.001
SE, %	77.1 ± 24.8	87.0 ± 17.3	0.019
SL, min	2.4 ± 2.4	2.1 ± 2.4	0.511
WASO, min	1.6 ± 4.0	1.6 ± 3.2	0.615
Stage R latency, min	14.0^§^	4.8 ± 1.1	0.221
N1, min	5.0 ± 3.4	8.1 ± 6.0	0.029
N2, min	6.6 ± 2.8	9.4 ± 4.7	0.014
N3, min	0.0 ± 0.1	4.9 ± 5.2	< 0.001
R, min	0.0 ± 0.2	0.1 ± 0.6	0.556
Subjective evaluation			
Nap time, min	18.7 ± 8.8	19.5 ± 11.4	0.867
SL, min	6.5 ± 6.0	7.1 ± 5.3	0.550
Depth of the nap^†^	2.5 ± 1.1	2.9 ± 1.1	0.252
Nap satisfaction^¶^	2.8 ± 0.8	2.8 ± 0.8	0.875

SE, sleep efficiency; SL, sleep latency; TRT, total recording time; TST, total sleep time; WASO, wake after sleep onset. ^§^Only one participant recorded. ^†^1: light, 5: deep. ^¶^1: poor, 4: good.

### Experiment 1: Post-nap task performance

#### Correct responses on the digit-symbol substitution test (DSST)

**Fig. 2:**
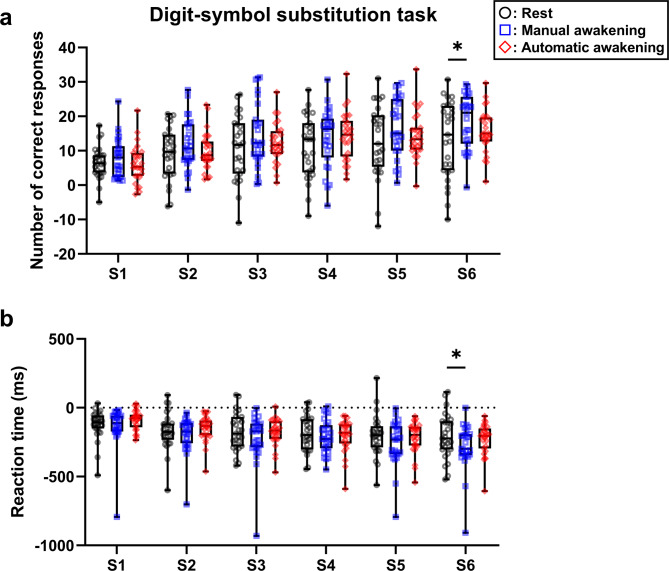
Task performance during post-nap sessions on digit-symbol substitution. (**a**) The number of correct responses results. (**b**) The reaction time results. The changes from baseline were calculated as the mean value of the three task bouts in one session and subtracted from the baseline to the post-nap session. Boxes show the interquartile ranges from 25% to 75% and medians. Whiskers indicate the range between the maximum and minimum. Data points are plotted with black circular dots in the rest group, blue squares in the manual awakening group, and red diamond-shaped dots in the automatic awakening group. *p < 0.05. S, session.

In the primary analysis, the automatic awakening group exhibited no significant difference in DSST performance in any of the post-nap sessions compared to the rest group (Fig. 2a). In the key secondary analysis, the manual awakening group had a significantly increased number of correct responses compared to that of the rest group in session 6 (S6; Fig. 2a, *p* = 0.029). Intergroup comparisons using the maximum contrast method showed an improvement in the manual—automatic—rest order in S6 (*p* = 0.028). No significant intergroup difference was observed in the other post-nap sessions.

#### DSST reaction time (RT)

In S6, the manual awakening group had significantly shortened RTs compared to that of the rest group (Fig. 2b, *p* = 0.023). Intergroup comparisons showed a manual—automatic—rest order of improvement in S6 (*p* = 0.022). The other sessions showed no significant intergroup differences.

#### Number of visual detection task (VDT) missed

No significant intergroup differences were noted in the number of VDT misses in any session after napping (see Supplementary Fig. S1a online). Intergroup comparisons revealed no significant differences across sessions.

#### VDT RT

No significant intergroup differences were observed in any session after napping (see Supplementary Fig. S1b online). Intergroup comparisons revealed no significant differences across sessions.

### Experiment 1: slow eye movement (SEM) evaluation

**Fig. 3:**
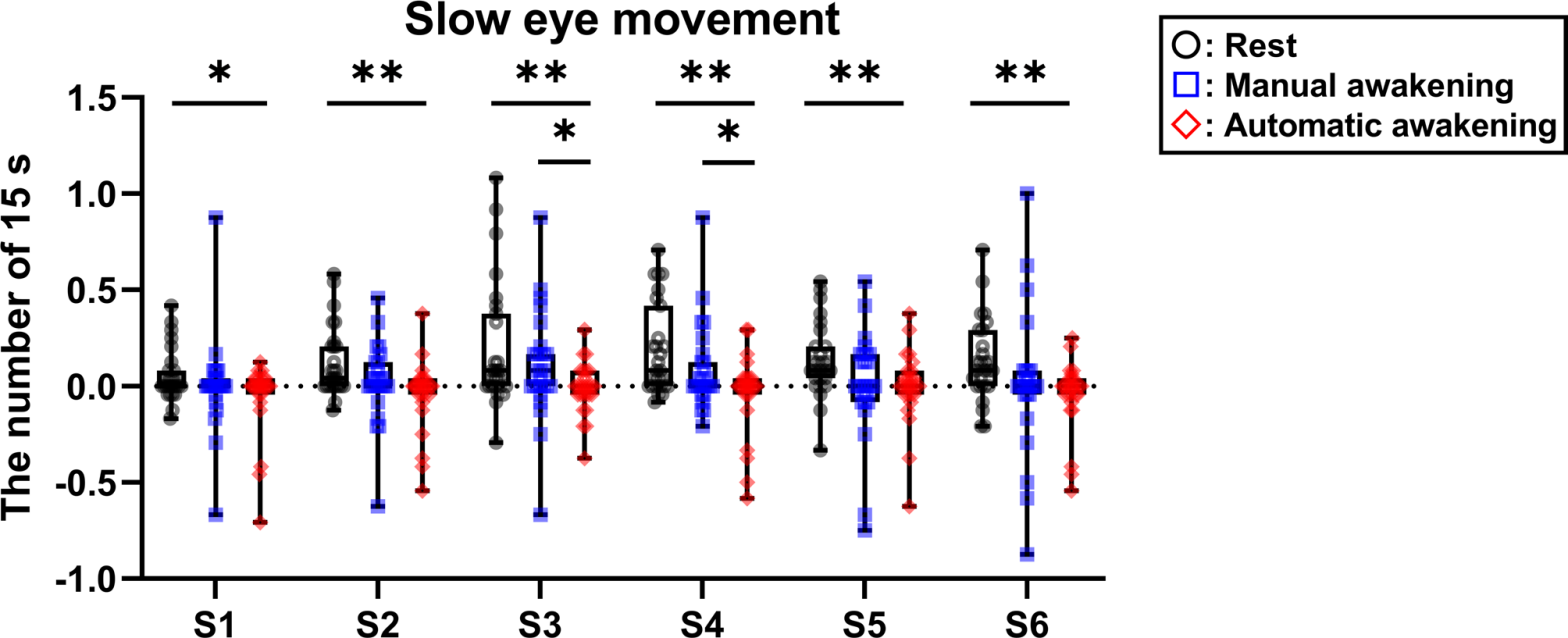
Objective sleepiness evaluation using the number of slow eye movement during the post-nap visual detection task. The slow eye movement number was counted in the 15-s epoch during the 2 min of the visual detection task. The changes from baseline were calculated as the mean value of the three task bouts in one session and subtracted from the baseline to the post-nap session. Boxes show the interquartile ranges from 25% to 75% and medians. Whiskers indicate the range between the maximum and minimum. Data points are plotted with black circular dots in the rest group, blue squares in the manual awakening group, and red diamond-shaped dots in the automatic awakening group. *p < 0.05, **p < 0.01. S, session.

The number of SEMs significantly decreased in the automatic awakening group compared to that in the rest group during all post-nap sessions (Fig. 3). There was no significant difference between the manual awakening and rest groups (Fig. 3). The number of SEMs significantly decreased in the automatic awakening group compared to that in the manual awakening group from S3 to S4 (Fig. 3). The maximum contrast method indicated that the reduction in the number of SEMs was significantly equivalent between the manual and automatic awakening groups from S2 to S6. No significant intergroup difference was observed in S1.

### Experiment 1: subjective sleepiness and fatigue evaluations

#### Karolinska sleepiness scale (KSS) for subjective sleepiness evaluation

**Fig. 4:**
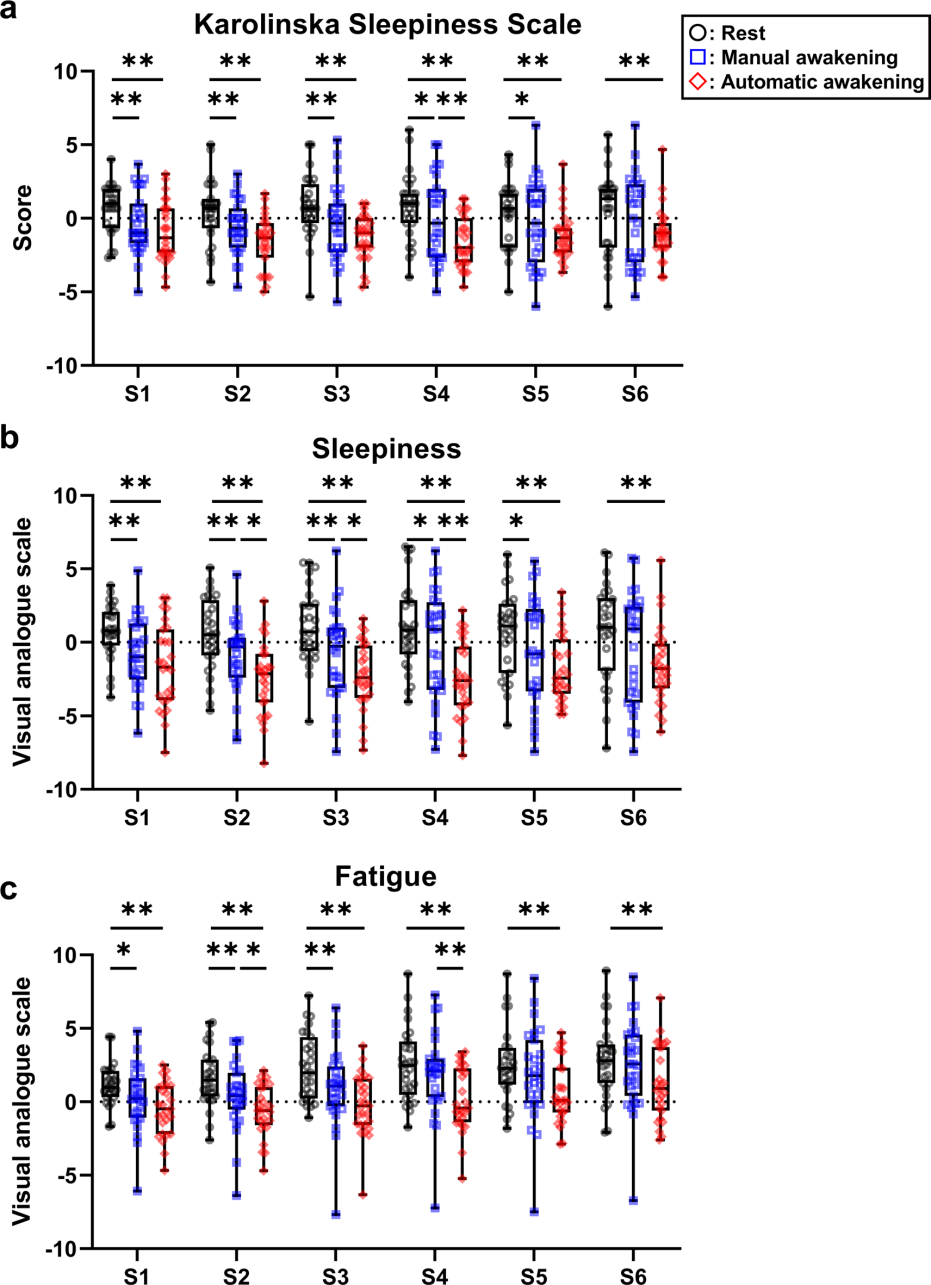
Subjective evaluation of sleepiness and fatigue during post-nap sessions. (**a**) Subjective sleepiness evaluation using Karolinska Sleepiness Scale. (**b**) Subjective sleepiness using visual analogue scale. (**c**) Subjective fatigue using visual analogue scale. The changes from baseline were calculated as the mean value of the three task bouts in one session and subtracted from the baseline to the post-nap session. Boxes show the interquartile ranges from 25% to 75% and medians. Whiskers indicate the range between the maximum and minimum. Data points are plotted with black circular dots in the rest group, blue squares in the manual awakening group, and red diamond-shaped dots in the automatic awakening group. *p < 0.05, **p < 0.01. S, session.

The KSS score was significantly lower (i.e., subjective sleepiness improved) in the automatic awakening group than in the rest group across all post-nap sessions (Fig. 4a). The manual awakening group showed significantly improved sleepiness compared to the rest group from S1 to S5 (Fig. 4a). The automatic awakening group showed significantly decreased sleepiness compared to the manual awakening group during S4 (Fig. 4a). The maximum contrast method showed that the KSS scores of the manual and automatic awakening groups were significantly equivalent in all post-nap sessions.

#### Visual analogue scale (VAS) for subjective sleepiness evaluation

The results of the VAS were significantly reduced; that is, subjective sleepiness improved significantly more in the automatic awakening group than in the rest group in all sessions (Fig. 4b). Sleepiness significantly improved in the manual awakening group, compared to the rest group, from S1 to S5 (Fig. 4b). The automatic awakening group showed significantly decreased sleepiness compared to the manual awakening group from S2 to S4 (Fig. 4b). Furthermore, group comparisons indicated that the manual and automatic awakening groups were significantly equivalent across all post-nap sessions.

#### VAS for subjective fatigue evaluation

The VAS scores were significantly reduced; that is, subjective fatigue improved significantly more in the automatic awakening group than in the rest group in all post-nap sessions (Fig. 4c). Compared to the rest group, fatigue significantly improved in the manual awakening group from S1 to S3 (Fig. 4c). The automatic awakening group showed significantly decreased fatigue compared to the manual awakening group during S2 and S4, respectively (Fig. 4c). The maximum contrast method indicated that the manual and automatic awakening groups were significantly equivalent throughout the post-nap sessions.

### Experiment 2: Second-generation automatic N2 detection system

The participants’ characteristics and sleep parameters are summarized in Supplementary Tables S2 and S3. No adverse events were reported.

For the first N2 that reached N3 without awakening or that followed ≥ 9 min of stable sleep, the system’s N2-detection accuracy ± 6 min was 36.0%. When the range was expanded to ± 16 min, the accuracy reached 80.0%. The accuracy of the first N2, calculated using the AASM criteria^31^, was 58.0% in ± 6 min. When the range was expanded to ± 10 min, the accuracy reached 80.0%. Contrary to expectations, the detection of N2-defined AASM criteria^31^ was more successful for the first N2. Additionally, the apnea-hypopnea index (AHI)^31^ was obtained using PSG. The accuracy was calculated for 38 participants in the low AHI group (AHI < 15/h), and showed 65.8% accuracy in ± 6 min; the accuracy reached 81.6% when using ± 8 min, which was close to the target range for participants with fewer respiratory events.

## Discussion

Similar to manual awakening, the automatic awakening system improved subjective sleepiness and fatigue. Though the number of SEMs decreased in the automatic awakening group, compared to the rest group, there was no difference in SEM occurrence between the manual awakening and rest groups. In contrast, napping with an automatic awakening system failed to improve DSST performance whereas napping with manual awakening improved DSST performance. The first-generation automatic awakening system differed from that of manual awakening, and because of its low accuracy, we increased the training data. This second-generation system improved the accuracy of the first N2 detection, especially when the participants were restricted to those with fewer respiratory events.

In Experiment 1, discrepancies were observed between the measured parameters. Only the manual awakening group showed improved DSST performance. The SEMs decreased most effectively in the automatic awakening group. Subjective sleepiness and fatigue improved in both napping groups; however, the effects persisted longer in the automatic awakening group. Based on this study’s results, we could not clarify the physiological significance of the periods wherein the discrepancies occurred. Although the cause of this discrepancy is unknown from the present results, we consider the following possibilities. First, it may be derived from the difference in sleep content between the two groups. Previous studies have also reported discrepancies between subjective and objective sleepiness assessments under sleep deprivation or nap experiments^32–35^. In this study, sleep duration was longer in the automatic awakening group than in the manual awakening group, resulting in the presence of N3 sleep, a putative marker of sleep pressure^36^. Previous studies have shown associations between sleep pressure and increased subjective sleepiness, subjective fatigue, and SEMs^32–34,37,38^. Napping in the automatic awakening group may have reduced sleep pressure, leading to improved subjective evaluations and fewer SEMs. It is also possible that sleep inertia induced by SWS^20^ might have inhibited performance improvement. Therefore, the divergence in sleep contents between the two groups may have induced differences in sleep pressure and sleep inertia upon awakening. Second, in the manual awakening group, the sleep length was constant, from N2 to 9 min, whereas in the automatic awakening group, it varied due to the low accuracy of N2 detection. This inter-individual variation in nap length may have resulted in dispersion in the effects of napping, which may have prevented performance improvement.

In the manual awakening group, this study partially replicated previous findings on cognitive performance^21,27^. The DSST improved in post-nap sessions 4–6 (S2 in the study) in the N2 nap condition compared to the rest group^27^. Despite the delayed task-improvement timing (S6 in the study), we replicated the experiment of Hayashi et al.^27^ for DSST performance. In contrast to a previous study^27^, there were no significant intergroup differences in VDT misses throughout the post-nap sessions in this study. The VDT RT results were similar to those of a previous study^27^, with no changes between conditions. Although the timing of the appearance of N2 differed among individuals, the TST in the manual awakening group may be comparable to the 10-min nap^21^. Brooks and Lack have reported that a 10-min nap improved correct responses on the DSST compared to rest at 35, 95, and 155 min post-nap^21^. The task-improvement timing, S6 in the study, was considered between 95 and 155 min^21^. Therefore, awakening from a nap 9 min after N2 could partially improve performance, even though the timing was delayed compared to previous studies^21,27^.

In contrast to a previous study^27^, the number of SEMs showed no decrease in the manual awakening group compared to the rest group. Several differences in design between the previous^27^ and present studies may have contributed to this difference. Hayashi et al. (2005) used a repeated-measures design (no nap, nap awakened 5 min after N1, and nap awakened 3 min after N2)^27^, whereas the present study used a parallel-group comparative design. Hayashi et al. (2005) focused on young participants aged 19–24 years^27^, whereas the present study examined older participants (mean age 33.6 years). Finally, in Hayashi *et al.’s* (2005) experiment^27^, the participants had a 2-h partial sleep deprivation before the experiment, while the current study did not. Therefore, it is possible that the participants’ pre-nap baseline SEMs were lower in the current study due to usual subjective sleepiness with no sleep deprivation; the number of post-nap SEMs may not have significantly decreased due to a floor effect. However, the reason for this discrepancy could not be determined.

Consistent with prior studies^21,27,35^, subjective sleepiness and fatigue in the manual awakening group decreased immediately and continuously after napping, which indicates the restorative effect of napping. Similar to previous studies^27,35^, this study indicates that 9-min naps after N2 are more effective and sensitive regarding subjective sleepiness/fatigue, rather than cognitive performance. Manual awakening produced little N3, consistent with previous studies^21,27,35^, which may have led to the exclusion of the effects of sleep inertia. The present study demonstrated the usefulness of a 9-min nap after N2 for subjective sleepiness and fatigue.

No post-nap improvement in the task performance was observed in the automatic awakening group. Hayashi et al. (2005) reported the absence of N3 under N2 nap condition^27^. In contrast, the average time from N2 in the offline PSG analysis to the alarm was 19.6 min in the automatic awakening group, which was longer than the optimal length of 9 min after N2^28^. As a result, the proportion of those who reached N3 was 63.0%, and the average N3 duration was 4.9 min in the automatic awakening group. A previous study showed some temporary sleep inertia effects in the 20-min nap condition^21^ that may be comparable to the sleep duration in the autonomic awakening group. For example, the N3 duration (sleep stage 3 + 4) was 5.7 min in the previous study^21^ and 4.9 min in the current study. Therefore, the longer nap length in the automatic awakening group may have conferred sleep inertia owing to SWS, which may have interfered with the performance-improving effects of the nap. However, contrary to the previous study^21^, the effect of sleep inertia on the automatic awakening group was not apparent at earlier timepoints. The variation in nap length may have dispersed the effect of sleep inertia after awakening. In addition to sleep inertia, inter-individual variability in sleep duration may be one possible factor that inhibited performance improvement. Since TST in the automatic awakening group was more variable, the inter-individual variability could have masked group-level effects. Nevertheless, this cause could not be clarified in the present study.

In Experiment 1, the performance of the BF meter for detecting N2 was relatively low. Furthermore, the 19.6 min from N2 detection to alarm using the system was longer than the target duration of 3–15 min after N2 exposure^27,28^. With a focus on post-nap task performance, future studies are required to increase the accuracy of N2 estimation using BF meters. We consider two reasons for the poor performance of N2 detection using the automatic system. First, though the present study was conducted in healthy adults, participants who were unaware of sleep disorders, such as obstructive sleep apnea (OSA) and periodic limb movements of sleep (PLMS), could have been included. Hemodynamic changes derived from heart rate fluctuations owing to OSA or PLMS^39,40^ may have interfered with the current N2-detection results. In the future, it will be necessary to ascertain interindividual variations in the N2-detection accuracy of the device among those with sleep disorders. Second, there may be room for improvement in the BF device design and automatic sleep-staging model. In particular, *n* = 81 training data were used in developing the automatic awakening system; however, additional training data, including data from participants with sleep disorders, may be required in future studies.

Based on this discussion, Experiment 2 was conducted. The second-generation system’s N2 detection accuracy was close to the target calculated only for participants with fewer respiratory events. This result may support the idea that sleep apnea could interfere with current N2 detection. Though there is a need to further improve accuracy, our results indicate the possibility of achieving the desired accuracy by adding training data and improving the model. For patients with severe respiratory events, using the system with the participant in the lateral position may be better for preventing respiratory events^41^. As the use of a body pillow increases the use of the lateral position^42^, it may be interesting to investigate whether the N2-detection accuracy in participants with OSA can be improved by using a pillow along with this automated system.

This study had some limitations. First, the accuracy of the N2 detection model warrants improvement. Due to the low accuracy, TST increased more in the automatic awakening group than in the manual awakening group. The differences in TST and sleep stages might have been confounding factors in comparison between the two groups. The accuracy of N2 estimation of BF can be increased by adding training data, individualizing N2 detection, or integrating biometrics such as temperature and electrodermal activity sensors. Second, the participant characteristics showed an intergroup difference in BMI between the rest and manual awakening groups; the cause for this difference is unclear because normal-weight participants were selected (inclusion criteria: BMI 18.5–24.9 kg/m^2^) and randomly allocated to the study groups. As few previous studies have examined the effects of BMI on napping, it may be necessary to study participants with various BMIs in the future to investigate the impact of BMI on napping. Third, the absence of a double-blind study design may have introduced participant expectations or placebo effects, especially in subjective evaluations. Fourth, the participants were limited to healthy adults. It is important to evaluate whether the accuracy of automatic awakening systems changes in patients with sleep disorders. Fifth, the study was conducted in a sleep laboratory. Future studies should evaluate the use of automatic awakening systems in real-world settings. Finally, for statistical tests, no adjustments were made for multiplicity except for the primary analysis; therefore, the results should be interpreted with caution.

In conclusion, napping with an automatic awakening system improved subjective sleepiness, SEMs, and subjective fatigue similar to that with manual awakening. Improving the accuracy of the N2 detection may lead to performance improvement which reaches that of manual awakening.

## Methods

### Experiment 1

#### Participants

The target number of participants was 81, with 27 participants per group, in a three-arm parallel-group study. The rationale for this sample size setting was based on that of Hayashi et al. (2005)^27^; in the previous study, the mean intergroup difference between the N2 nap and the rest groups of correct responses on the DSST at post-nap sessions 4–6 was 9.7, with an SD of 9.8. Therefore, the difference in the amount change of correct responses on the DSST between the automatic awakening and rest groups was estimated at nine and SD at 11, with a significance level of 5% and power of 80%. The required number of participants per group in the primary analysis was 25 participants per group. The number of samples per group was set to 27 to allow for dropouts. For the key secondary analysis, an equal number of participants were recruited for the manual awakening group, with the total target number of participants being 81.

The participants joined the screening using the Internet Bulletin Board. The examiner obtained documented consent from all participants. The inclusion criteria were as follows: (i) age 20–59 years, (ii) literacy in Japanese, (iii) ability to nap in an experiment room, and (iv) not currently undergoing treatment for sleep disorders. Exclusion criteria were as follows: (i) irregular lifestyle with bedtimes outside the range of 21:00 to 1:00, waking up before or after 6:00 to 9:00, or more or less than seven to nine hours of sleep each night; (ii) BMI < 18.5 or ≥ 25.0 kg/m^2^; (iii) history of night-shift work after 22:00; (iv) travel history to a country with more than three hours difference with Japan in the last 3 months; (v) habitual alcohol intake (≥ 40 g of alcohol at least twice a week); (vi) smoker status; (vii) caffeine intake ≥ 400 mg per day; (viii) pregnant or breastfeeding; (ix) history of treatment for psychiatric disorders or sleep disorders, or currently have symptoms for which are treated; (x) participants with the Pittsburgh Sleep Quality Index (PSQI) scores ≥ 6^43,44^; xi) participants who scored Morningness–Eveningness Questionnaire (MEQ) ≤ 30 points (definitely morning type) or ≥ 70 points (definitely evening type)^45,46^; xii) claustrophobia; xiii) those with illnesses or a history of diseases have the potential for sudden change. Of those who participated in the screening (*n* = 87, 30.7 ± 12.7 years, 51 female), 12 persons unmet for PSQI (one person excluded because of also BMI), and three persons unmet the criteria for MEQ (one person excluded because of also BMI). Three participants withdrew their consent. Nine individuals were unable to schedule the experiments. Therefore, 60 participants were included. As the Internet Bulletin Board alone provided insufficient participants, we asked the recruiting company to introduce people who fulfilled the experimental requirements (*n* = 21, 40.7 ± 10.0 years, 8 female). Participants provided written informed consent after receiving verbal and written explanations of the experiment. Finally, 81 healthy adults participated (Table 1). The Ethical Committee of the University of Tsukuba Hospital approved this research (approval number: R03-094), and the study was performed in accordance with the principles of the Declaration of Helsinki. This study was registered in the University Hospital Medical Information Network Clinical Trials Registry (04/10/2021, UMIN000045658 [https://center6.umin.ac.jp/cgi-open-bin/ctr_e/ctr_view.cgi?recptno=R000052112]).

#### Experimental protocol

In this controlled, randomized, non-blinded, parallel-group comparative study, 27 participants each were randomly allocated to automatic awakening, manual awakening, and rest groups. Each group was randomly assigned to numbers 1–81, and the participants who passed the screening were assigned the condition in order by number. Before the experiment, participants were examined for their sleep/wake cycle using a Fitbit Charge 3 (Fitbit, Inc., CA, USA) and a sleep log for a week.

Alcohol, medication, tobacco, and caffeine intake were prohibited from 11:00 on the day before the experiment. Alcohol, medication, napping, excessive exercise, tobacco, and caffeine consumption were not allowed from the time of waking on the day of the experiment until the end of the experiment. Personal electronic devices, such as smartphones, turned off their power during the experiment.

**Fig. 5:**
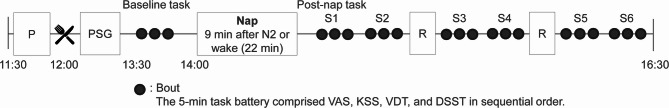
Study protocol. Participants practiced the tasks (P in the figure) from 11:30. After lunch, the participants were fitted using polysomnography (PSG). A baseline task was performed for 15 min (one bout of 5 min duration × three times), starting at 13:30. After the baseline task, the participants were told whether they would perform the nap or rest condition and were unclarified whether they would be automatically or manually awakened. The automatic/manual awakening group was placed on a blood flow meter and napped at 14:00. They were awakened when N2 appeared, and 9 min had elapsed. The rest group remained awake for 22 min. The task was repeated after napping or resting. The task was carried out for 15 min (three bouts) over six sessions. KSS, Karolinska Sleepiness Scale; P, practice; PSG, attachment to polysomnographic sensors; R, 15-min rest; S, session; VAS, visual analogue scale; VDT, visual detection task; DSST, digit-symbol substitution test.

The experimental protocol on the day of the experiment is shown in Fig. 5. The participants arrived at the sleep laboratory in the International Institute for Integrative Sleep Medicine (WPI-IIIS) at 11:30 and practiced the tasks. The experimenters provided the participants with lunch and controlled their calorie intake based on weight. Calories were individually standardized based on the estimated energy requirement for Japanese adults, assuming a physical activity level of 1.5^47^. After lunch, the PSG were attached to the participants. From 13:30 to 13:45, a 15-min (three 5-min task bouts) task was conducted as a pre-nap session. Each 5-min task battery included the VAS, KSS, VDT, and DSST. Participants were informed of their group assignment, nap or rest, prior to the nap. However, those in the nap group were not made aware of their sleep condition until the experiment was completed. After wearing the BF device for both nap groups, the lights-out was recorded at 14:00. In the automatic awakening group, BF measurements were started at least 8 min before lights-out because the system required time for the model to begin N2 detection. In the nap with automatic awakening condition, participants were awakened 9 min from N2, as judged by the automatic awakening system. The participants’ PSGs were monitored by a scorer during a nap in the manual awakening condition. When 9 min had elapsed from the first occurrence of a sleep spindle or K-complex^27^, the alarm manually awakened the participants. The manual awakening group was manually awakened 30 min after lights-out if they could not fall asleep or did not reach N2. In the automatic awakening group, if the alarm did not go off within 30 min after lights-out, participants were manually awakened as well. All these participants were included in the analysis. In the rest group, the participants sat and watched a Digital Versatile Disc film on a laptop for 22 min. The time of 22 min was determined based on another nap experiment conducted in our laboratory (R03-094). In the previous experiment, the average time that elapsed for 9 min without awakening after the first N2 was 21.6 min, consequently, we set 22 min as the rest condition. After the nap, the napping group completed a questionnaire regarding their subjective sleep assessment. The questionnaire is similar to that used by Hayashi et al. (2005)^27^. The questionnaire contained four questions: “How long do you think the nap lasted?” “How long did it take you to fall asleep?” and “Depth of sleep: select a number from 1 to 5 with one being light and five being deep”; and “Satisfaction with your nap: select a number from 1 to 4 with one being poor and four being good.” The task was then conducted in sets of 15 min (3 times 5 min) for six sessions (S1-6). A 15-min break was included between S2 and S3 and between S4 and S5. Participants in the napping group were asked to answer a questionnaire about adverse events when all tasks were completed. The experiment was terminated after removal of the PSG.

#### Experimental room setting

The PSG, smartphone in the automatic awakening system, and task laptops were time-synchronized with radio-controlled clocks. During the experiment, the lighting in the room was less than 100 lx. The nap group napped with the lights off, while the rest group kept the lights on during the rest period. The room temperature and humidity were monitored using a thermo-hygrometer (Testo 175H1 Temperature and Humidity Data Logger, Testo SE & Co. KGaA., Titisee-Neustadt, Germany). Air conditioning and a dehumidifier were set to ensure the room temperature and humidity were at levels that the participants found comfortable (temperature 23.2 ± 1.0℃, humidity 43.7 ± 12.2%).

#### Task performance

Performance before and after napping was evaluated using the VDT and DSST, as in a previous study^27^. First, the target number (0–9) was presented on the monitor for 1 s. After 4 s, various numbers (0–9) were presented randomly for 200 ms each^27^. When the target number appeared on the screen, participants pressed the button immediately. One hundred trials were presented per session, during which the target was presented 10 times^27^. We evaluated the RT and number of misses for the VDT. The DSST measures cognitive processing speed^48,49^. At the start of the task, numbers 1–9 and symbols were displayed at the top of the monitor. Subsequently, a symbol is presented at the bottom of the monitor. When the symbol appeared, the participants had to press the keyboard with the number corresponding to the symbol as soon as possible. We evaluated the RT and number of correct responses for analysis of the DSST.

#### Subjective evaluation of sleepiness and fatigue

The VAS was used to evaluate subjective sleepiness and fatigue, as described by Hayashi et al. (2005)^27^. Sleepiness and fatigue were scored using a 10-mm VAS. The KSS was used to evaluate subjective sleepiness^50,51^.

#### Sleep evaluation

PSG using the PSG-1100 (Nihon Kohden, Tokyo, Japan) was performed according to the AASM criteria and consisted of electroencephalography (F3-M2, F4-M1, C3-M2, C4-M1, O1-M2, and O2-M1), chin electromyography (EMG), electrooculography (EOG), electrocardiography, airflow, and nasal pressure^31^. PSG was recorded at a sampling frequency of 250 Hz. PSG was scored by a registered polysomnographic technologist using the AASM criteria^31^. The following sleep parameters were calculated: TST, sleep latency, SE, wake after sleep onset, N1, N2, N3, and R duration, and stage R latency^31^. To assess the efficacy of N2 detection, the time of first appearance of the spindle and K-complex as the N2 detection time was examined using offline PSG analysis. Furthermore, the time from the appearance of these N2 indices until the alarm was switched off was calculated.

#### SEM analysis

SEM analysis was performed according to the method described by Hayashi et al. (2005)^27^ to evaluate objective sleepiness^38,50,52^. The number of 15-s epochs in which any SEMs occurred during the 2 min of VDT was counted using EOG^27^. Eye movements with amplitudes greater than 50 µV and durations more than 1 s were considered SEMs^27,53^. As AASM defines SEM as conjugate, regular, sinusoidal eye movements with an initial deflection and lasting > 0.5 s of initial eye movement deflection^31^, rapid eye movements were differentiated from SEMs using the AASM criteria^31^.

#### Automatic awakening system using the BF sensor

An earphone-shaped sensor (KYOCERA, Kyoto, Japan) with a 39-Hz sampling rate was placed on the concha of the right ear to measure the BF and 3-axis acceleration^30^. The BF measurement was based on the laser Doppler method. This provides a continuous estimate of skin BF restricted to skin microcirculation and can be performed in different areas and surfaces. Customized sensors can simultaneously estimate the pulse rate variability and BF, thus providing more information than PPG devices^30^. The BF data were sent and preserved on a smartphone. From the smartphone application, the BF and 3-axis acceleration data are sent to the cloud, and the automatic awakening model in the cloud determines the participant’s sleep stage. If the system detected the first N2 timing, it set a wake-up alarm 9 min after this point on the smartphone. N2 timing was defined as the first N2 that reached N3 without awakening, or that was followed by ≥ 9 min of consecutive sleep. By determining N2 after ± 6 min and sounding an alarm after 9 min, the system enables the determination of optimal nap duration, which is 3–15 min after N2 (Fig. 1). The model for N2 detection was used as training data for sleep-stage scoring performed during a daytime napping experiment with 81 healthy adults, in which PSG and the BF meter assessments were conducted simultaneously (approval number: R04-037).

#### Statistical analysis

In the primary analysis, the difference in the change in the number of correct responses in the DSST at six time points (S1–6) after the nap between the rest and automatic awakening groups was assessed using a mixed-effects model for repeated measures (MMRM)^54^. Group comparisons were also performed at each session using the MMRM. The model was adjusted for groups, time points (categories), group × time point interactions, and baseline values. In the key secondary analysis, the three groups—rest, automatic awakening, and manual awakening—were compared using the same approach as the primary analysis. The maximum contrast method, which facilitates the collective evaluation of the relationships among groups and model selection^55^, was used for intergroup comparisons. Therefore, the results obtained using the maximum contrast method were prioritized to evaluate the hypothesis regarding the relationship among the three groups. The contrasts used in the analysis were (rest group, automatic awakening group, manual awakening group) = (− 1, − 1, 2), (− 2, 1, 1), (− 1, 0, 1). We assessed (1) manual best (− 1, − 1, 2), (2) automatic and manual are equivalent (− 2, 1, 1), or (3) manual-automatic-rest order (− 1, 0, 1). This hypothesis was substantiated if the maximum contrast method selected a contrast of (− 2, 1, 1) or (− 1, 0, 1). The group, time point (category), group × time point interaction, and baseline values were included in the model as fixed effects. An unstructured covariance structure was specified to account for inter-individual variation. The same analysis was conducted for other outcomes, including RTs for the DSST and VDT, the number of VDT misses, the number of SEMs, and the scores for KSS and VAS outcomes. Robust variance was used in the MMRM analysis of the SEM to handle data containing many zeros.

For continuous variables, differences among the three groups were tested with the one-way analysis of variance for normally distributed parameters or with the Kruskal–Wallis test for nonnormally distributed parameters, as appropriate. Categorical data were compared using the Fisher’s exact test. The Mann–Whitney *U* test was used to compare sleep stages in the napping group. The results were described by mean ± SD. To assess the equality of variances, Levene’s test was used. A significance level of 5% was considered for all analyses. Multiplicity for statistical tests was not adjusted, except for the primary analysis; therefore, the results should be interpreted with caution. Sample size determination and analyses were performed with SAS version 9.4 (SAS Institute, Cary, NC, USA).

We calculated the accuracy of N2 detection using BF as follows. The first N2 was defined as reaching N3 without awakening or followed by ≥ 9 min of consecutive sleep. N2 was set to avoid the influence of changes in the BF due to arousal. Additionally, the first N2 was also assessed according to the AASM criteria^31^. Accuracy (the number of correct responses/total number × 100) was calculated, assuming the correct response if the system detects the first N2 within ± 6 min or if the N2 was undetected both in the system and PSG.

### Experiment 2

#### Participants

Previous sleep device validation studies, such as those involving the Oura Ring and Fitbit with PSG, validated 40 and 53 participants, respectively^56,57^. Therefore, we used these two studies as references for validation, with a target sample size of 50 participants.

The selection criteria were almost the same as in Experiment 1, except that the age inclusion criterion was set to ≥ 20 years, and the BMI exclusion criterion was set to only those with BMI < 18.5 kg/m^2^ to obtain BF data from obese participants. In Experiment 2, the PSQI score was not used as an exclusion criterion. In addition to the exclusion criteria, we excluded participants with a history of experiments from which the training data were collected.

Twenty-four participants were recruited from the Internet Bulletin Board, and participated in the screening (24.8 ± 8.2 years, 13 female). Written and verbal explanations of the experiment were provided before screening, and written informed consent was obtained. Two participants were excluded because they had participated in previous experiments collecting training data, one was excluded because he had night shift work in the previous three months, and two were excluded because of their BMI. Seven participants could not schedule the experiments. Twelve individuals participated in the experiment. The recruitment company referred 38 participants (45.2 ± 10.2 years, 16 females). Finally, 50 participants participated (Supplementary Table S2). The Ethical Committee of the University of Tsukuba Hospital approved this research (R04-037), and the study was performed in accordance with the principles of the Declaration of Helsinki. This study was registered in the University Hospital Medical Information Network Clinical Trials Registry (03/10/2023, UMIN000052386 [https://center6.umin.ac.jp/cgi-open-bin/ctr_e/ctr_view.cgi?recptno=R000059803]).

#### Experimental protocol

Participants controlled their lifestyle the same as in Experiment 1 from the day before the experiment to the end of the experiment. In Experiment 2, the participants did not record their sleep-wake cycles using a Fitbit before the experiment. The participants arrived at the sleep laboratory in the WPI-IIIS at 12:00. Lunch was provided to the participants by experimenters using the same method as in Experiment 1. After lunch, the participants were fitted with a PSG (PSG-1100), which consisted of the same sensors as in Experiment 1, pulse oximeter, body position sensor, respiratory effort sensors, and leg EMG were added. They underwent simultaneous BF measurements and PSG during a 90-min nap from 14:30 to 16:00. BF measurements were started at least 7 min before lights-out because the system required time for sleep-stage determination. The automatic awakening system did not set any alarms in this experiment. Participants completed a questionnaire about the adverse events of the BF system after a nap.

#### Experimental room setting

The PSG and smartphone clocks were synchronized using a radio-controlled clock. The room temperature was set as a comfortable room temperature for the participant. Room temperature and humidity were monitored using a thermo-hygrometer (Testo 175H1 Temperature and humidity data logger; temperature 22.8 ± 0.7 °C, humidity 52.7 ± 4.2%).

#### Sleep evaluation

The same scorer in Experiment 1 scored the sleep stage, respiratory events, and PLMS, then calculated the sleep variables, AHI, and PLMS index^31^. We compared the first N2 timing in the log of the automatic awakening system and the PSG.

#### The automatic awakening system using the BF sensor

Unlike the first-generation device design, the second-generation BF meter (KYOCERA) was designed to be worn in both ears (a BF sensor in the right ear and 3-axial acceleration sensor in the left ear). The smartphone and the BF meter were connected via Bluetooth. An automatic N2 detection model was built into a smartphone application, allowing offline N2 detection. Data from 120 healthy adult participants during the naps were used as training data.

#### Statistical analysis

The accuracy of N2 detection was determined using the same methods as in Experiment 1.

## Supplementary Information

Below is the link to the electronic supplementary material.

Supplementary Material 1

## Data Availability

The datasets generated in this study are available from the corresponding author upon reasonable request.
