# Supplementary material for: Effects of optimal timed automatic awakening from a short daytime nap on cognitive performance, alertness, and fatigue
Source: Sci Rep. 2025 Oct 24;15:37228. doi: 10.1038/s41598-025-21008-3 (PMC12552439; doi:10.1038/s41598-025-21008-3)
Supplement: Supplementary file 1 — Supplementary Material 1 [file 41598_2025_21008_MOESM1_ESM.pdf]

Supplementary Information

Effects of Optimal Timed Automatic Awakening from a Short Daytime Nap on Cognitive Performance Alertness and Fatigue

Yoko Suzuki[1,+], Chihiro Suzuki[1,+], Yurina Suzuki[1], Fusae Kawana[1], Tomohiro Ohigashi[2], Kazushi Maruo[3], Takahiro Watanabe[4], Takashi Abe[1,*]

[1]International Institute for Integrative Sleep Medicine (WPI-IIIS), Tsukuba Institute for Advanced Research (TIAR), University of Tsukuba, Tsukuba, 305-0821, Japan

[2]Tsukuba Clinical Research & Development Organization, University of Tsukuba, Tsukuba, 305-8575, Japan

[3]Institute of Medicine, University of Tsukuba, Tsukuba, 305-8575, Japan

[4]KYOCERA Corporation, Kyoto, 612-8501, Japan

***Corresponding author**: Takashi Abe

WPI-IIIS, TIAR, University of Tsukuba, 1-2 Kasuga, Tsukuba, Ibaraki 305-0821, Japan

Email: abe.takashi.gp@u.tsukuba.ac.jp

[+]These authors contributed equally to this work.

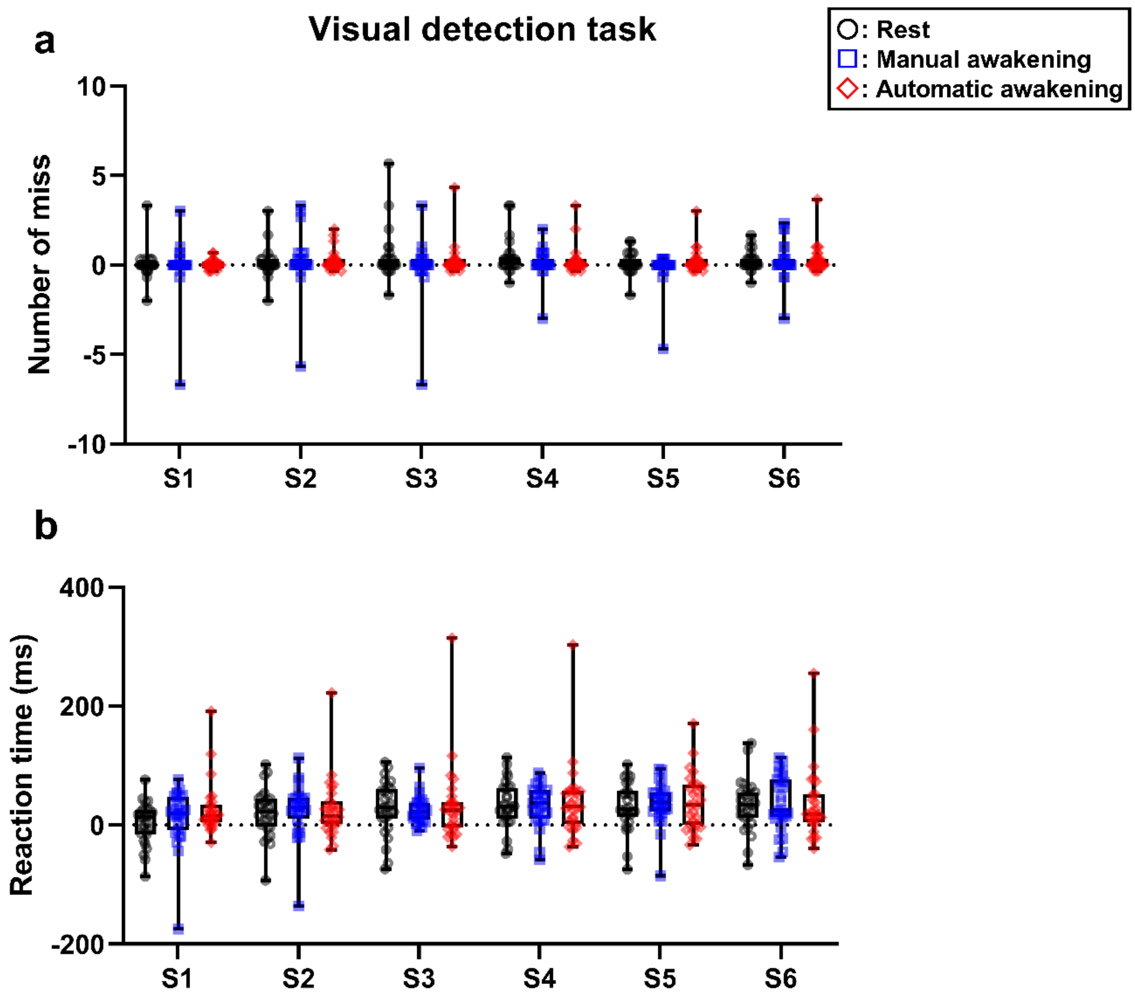

Supplementary Fig. S1. Task performance during post-nap sessions on visual detection task.
(a) The number of missed results. **(b)** The reaction time results. The changes from baseline were calculated as the mean value of the three task bouts in one session and subtracted from the baseline to the post-nap session. Boxes show the interquartile ranges from 25% to 75% and medians. Whiskers indicate the range between the maximum and minimum. Data points are plotted with black circular dots in the rest group, blue squares in the manual awakening group, and red diamond-shaped dots in the automatic awakening group.
S, session

Supplementary Table S1. Levene's test for sleep-stage scoring and subjective sleep evaluation in Experiment 1

	P-value
TRT, min	0.353
TST, min	<0.001
SE, %	0.309
SL, min	0.900
WASO, min	0.816
RL, min	NA §
N1, min	0.031
N2, min	0.031
N3, min	<0.001
R, min	0.122
Subjective evaluation	
Nap time, min	0.213
SL, min	0.808
Depth of the nap	0.403
Nap satisfaction	0.449

NA, not available; SE, sleep efficiency; SL, sleep latency; TRT, total recording time; TST, total sleep time; WASO, wake after sleep onset.

§Only one participant recorded in the manual awakening group.

Supplementary Table S2. Participants' characteristics in Experiment 2

Sex, n, male/female	26/24
Age, years	40.6±13.1
BMI, kg/m^2	22.7±3.3
MEQ-score	55.2±7.3
MEQ-type, n, Morning/Intermediate/Night	20/29/1
PSQI-score	3.9±2.0

Values are shown as mean±standard deviation.

BMI, body mass index; MEQ, Morningness–Eveningness Questionnaire; PSQI, Pittsburgh Sleep Quality Index

Supplementary Table S3. Sleep parameters in Experiment 2

TST, min	67.2±23.5
SE, %	74.8±26.2
SL, min	3.6±6.6
WASO, min	19.1±21.6
N1, min	22.2±11.9
N2, min	25.3±12.7
N3, min	16.7±14.5
R, min	3.0±5.7
Stage R latency, min	60.8±19.0
ArI, /h	44.0±27.4
AHI, /h	12.3±18.8
PLMSI, /h	3.6±9.5

Values are shown as mean±standard deviation.

AHI, apnea-hypopnea index; ArI, arousal index; PLMSI, periodic leg movement sleep index; SE, sleep efficiency; SL, sleep latency; TST, total sleep time; WASO, wake after sleep onset.
